# Directed deposition of silicon nanowires using neopentasilane as precursor and gold as catalyst

**DOI:** 10.3762/bjnano.3.62

**Published:** 2012-07-25

**Authors:** Britta Kämpken, Verena Wulf, Norbert Auner, Marcel Winhold, Michael Huth, Daniel Rhinow, Andreas Terfort

**Affiliations:** 1 Institute of Inorganic Chemistry, University of Frankfurt, Max-von-Laue-Straße 7, 60438 Frankfurt am Main, Germany; 2 Institute of Physics, University of Frankfurt, Max-von-Laue-Straße 1, 60438 Frankfurt am Main, Germany; 3 Department of Structural Biology, Max Planck Institute of Biophysics, Max-von-Laue-Straße 3, 60438 Frankfurt am Main, Germany

**Keywords:** chemical vapor deposition, gold, nanoparticle, patterning, radiation-induced nanostructures, vapor-liquid-solid mechanism

## Abstract

In this work the applicability of neopentasilane (Si(SiH_3_)_4_) as a precursor for the formation of silicon nanowires by using gold nanoparticles as a catalyst has been explored. The growth proceeds via the formation of liquid gold/silicon alloy droplets, which excrete the silicon nanowires upon continued decomposition of the precursor. This mechanism determines the diameter of the Si nanowires. Different sources for the gold nanoparticles have been tested: the spontaneous dewetting of gold films, thermally annealed gold films, deposition of preformed gold nanoparticles, and the use of “liquid bright gold”, a material historically used for the gilding of porcelain and glass. The latter does not only form gold nanoparticles when deposited as a thin film and thermally annealed, but can also be patterned by using UV irradiation, providing access to laterally structured layers of silicon nanowires.

## Introduction

One of the goals of nanoscience is the development of new materials. Nanowires offer unique insights into low-dimensional physics [1] and could play an important role as building blocks for nanosized devices [2]. Especially semiconducting nanowires can be usefully applied in the fields of biosensors [3] and chemical sensors [4], nanoelectronics [5], photonics [6] and photovoltaics [7]. In this context, it is important to be able to control parameters such as the diameter and length of the nanowires, as well as their localization [8]. Various techniques have been used in order to produce nanosized wires (NW) of silicon including thermal evaporation [9], molecular beam epitaxy [10], laser ablation [11], chemical vapor deposition (CVD) [12] and CVD in combination with the vapor–liquid–solid (VLS) method [13]. In the VLS mechanism, small solid metal particles catalyze the decomposition of the vaporous silicon precursor, forming a liquid Si–metal alloy. As more of the precursor is added to the system, crystalline silicon NWs are excreted from the alloy due to oversaturation. One effect of this mechanism is that the diameter of the NW is directly correlated to the particle size of the catalytic metal [14].

The most frequently used catalytic metal is gold [15] although other metals are known to catalyze the growth of silicon NWs as well [16–18]. If gold is used as a catalyst, e.g., by deposition of Au nanoparticles on the substrate, the VLS-growth process is reported to start above the eutectic point of the silicon–gold alloy at 363 °C. However, the best results are achieved at temperatures of 450 °C or higher [19]. Typical deposition methods for the metal include sputtering [20], or its adsorption in the form of nanoclusters [21] or nanoparticles [22]. The sputtering process requires no further treatment of the substrate before the VLS process. In contrast to this, organic molecules typically participate in the adsorption process of nanoparticles. Firstly, the nanoparticles themselves are almost always coated with a stabilizer to prevent agglomeration [23]. Secondly, the deposition process of nanoparticles often requires other reagents, e.g., for micelle nanolithography or chemisorption at surface-attached organic monolayers [24–25]. These organic additives (stabilizer/monolayer) might disturb the growth process of the silicon NWs and lead to contaminations, thus they need to be removed after the adsorption step in order to permit the undisturbed growth of silicon NWs. Depending on the nature of the stabilizer as well as the monolayer, positive results have been reported by simple thermolysis [26] or treatment of the substrates with either hydrogen or oxygen plasma [27]. An alternative way to prepare nanoparticles on surfaces was reported by He et al. [28]. They achieved the formation of gold nanoparticles on oxidic substrates by annealing of sputtered gold films. Variation of the thickness of the sputtered layer results in nanoparticles with a variety of sizes. Since the method is also applicable to other metals [29], it could prove to be a versatile way to create well-defined silicon NWs on metal-sputtered surfaces.

For the formation of silicon NWs by the CVD–VLS process, mainly monosilanes such as silane (SiH_4_) or tetrachlorosilane (SiCl_4_) have been used as starting materials due to their low cost and commercial accessibility [30–32]. Of these, silane bears the significant disadvantage of being a highly pyrophoric material, which upon contact with air immediately explodes, thus posing severe danger in the manufacturing process [33–34]. Tetrachlorosilane on the other hand is much safer but requires a reducing agent, such as hydrogen, for the formation of elemental silicon [35]. It also bears the possibility of contaminating the deposited silicon with chlorine atoms, which will significantly change the conduction behavior of the Si NWs. Different precursors such as octachlorotrisilane [36] and disilane [37] have similar properties to their monomeric analogues, although both of these precursors are reported to have an exceptionally high growth rate compared to their analogous monosilanes. This is due to the fact that the dissociation energy of the Si–Si bond is relatively small and can be supplied by thermal activation [38–39].

We herein report the first usage of neopentasilane (Si(SiH_3_)_4_, NPS) as a precursor for the growth of silicon NWs. NPS has a much higher silicon content (92 mass %) than most other silicon precursors. It contains four Si–Si bonds, so high growth rates can be expected [40]. Additionally the molecule contains no chlorine or other potentially contaminating atoms. At room temperature it is a liquid with a reasonable vapor pressure of 20 mbar [41]. In this work, we compare the growth of silicon NWs from NPS using three kinds of gold catalysts: Firstly, sputtered gold films without and with annealing, and secondly, preformed gold nanoparticles chemisorbed wet-chemically onto suitable substrates. Additionally, an unusual gold precursor, “liquid bright gold”, is spin coated onto a substrate and activated by an annealing step. We demonstrate how the different nature of the gold catalyst as well as the deposition temperature (375 versus 650 °C) changes the outcome of the growth. In addition, we will demonstrate that the “liquid bright gold” can be patterned by irradiation with UV light, which in turn results in localized deposition of the silicon NWs.

## Results and Discussion

### Sputtered gold films as catalyst

The first series of experiments was performed with Si[111] and borosilicate glass substrates. Borosilicate glass was used in this case to prove that the silicon in the grown nanowires originated from the precursor and not from the substrate. A uniform, thin layer of gold was sputtered onto the substrates with a sputtering time of one minute, corresponding to a thickness of about 10 nm. After transfer of these substrates into a tube reactor, NPS was carried into the reactor by a stream of argon.

**Figure 1:**
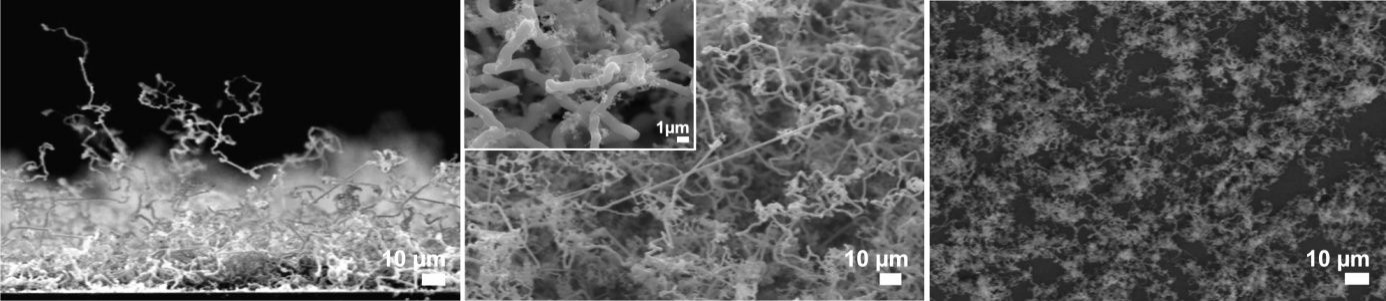
SEM images of NWs grown on gold-sputtered Si[111] exposed to NPS vapor at 375 °C for 1 h (left: side view; center: top view) and 30 min (right: top view). In the insert of the center view, the smaller, branching structures on the NWs become visible.

At a reaction temperature of 375 °C silicon NWs formed within a typical reaction time of 1 h. SEM images of the NWs show a thickness of 1 µm and varying length of up to several 100 µm (Figure 1, left and center).

**Figure 2:**
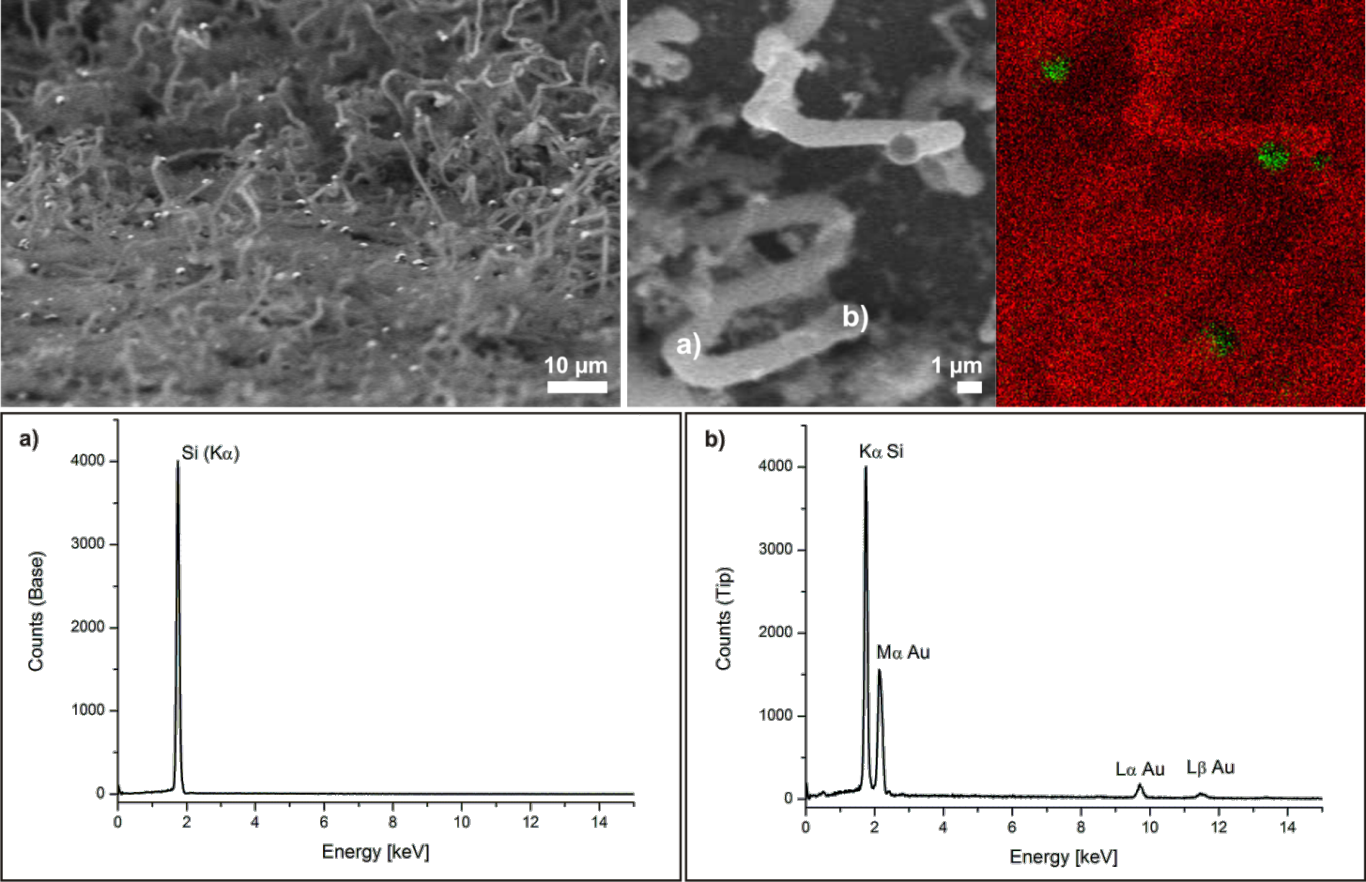
Analysis of NWs grown on gold-sputtered Si[111] for 1 h at 375 °C. Top left: Backscattered-electron image. The bright spots at the tips indicate the presence of a high-*z* material (gold). Top right: Composite EDX mapping image, green = gold, red = silicon. Bottom: EDX spectra obtained (a) at the base, (b) at the tip of the NWs.

The NWs do not grow evenly but are buckled in multiple directions, which presumably indicates that the catalytically active gold nanoparticles are of irregular shape and thus excrete the silicon nanowires in different directions. Nevertheless, several single NWs growing in a straight fashion over several hundreds of micrometers on the surface of the NW layer could be observed. The surfaces of the formed NWs are covered with smaller structures indicating that the NWs may still contain gold atoms, which catalyze the “branching” (Figure 1, insert in center image). Shortening the reaction time to 30 min resulted in a loosely packed layer of shorter NWs (Figure 1, right). Backscattered-electron images and EDX mapping of the NWs show that their bases as well as their bodies basically consist of pure silicon, whereas their tips are enriched with gold (Figure 2). These findings support the suggested VLS mechanism.

**Figure 3:**
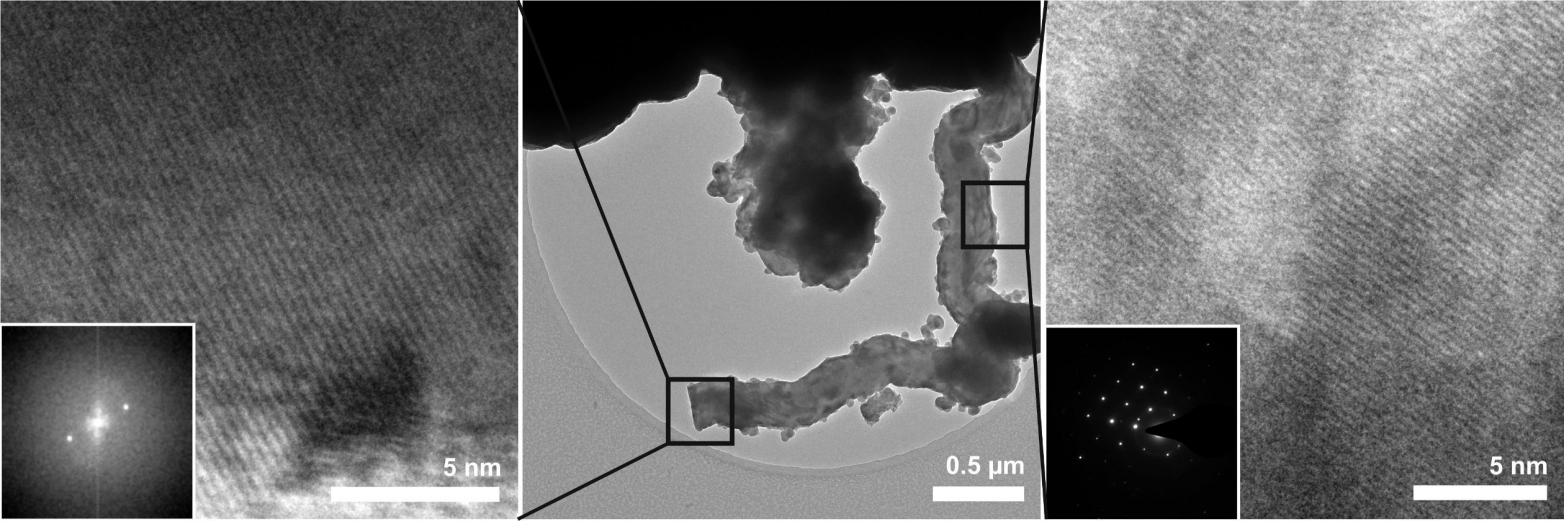
TEM images of NWs grown on gold-sputtered Si[111] for 1 h at 375 °C. The FFT of the HRTEM image and the SAED pattern indicate the crystallinity of the Si NWs.

To learn about the structure of the NWs, additional analysis by TEM was carried out. The TEM measurements confirm the buckled structure as well as the branched surface of the NWs (Figure 3, center). The HRTEM measurements show that the NWs are crystalline. The lattice constant of 3.2 Å, which can be seen in the fast Fourier transformed (FFT) image and the selected-area electron diffraction (SAED) pattern, as well as the hexagonal pattern visible in the latter indicate the presence of the cubic Si lattice.

The influence of the gas flow on the nanowire deposition was investigated by varying it between 0.1 and 1.0 L/min. In all cases NW growth was observed. At the lower flow limit, the length and diameter of the individual NWs resembled those of the NWs in the previously mentioned experiments; however, the substrate was not completely covered with NWs but rather showed only sparsely distributed, small NW islands. These results were similar to the ones obtained with a shortened reaction time and can in both cases be explained by the relatively small amount of precursor being carried into the system. At the upper limit of the gas flow, the NWs grew efficiently, covering most of the substrate. Nevertheless, a large amount of precursor seemed to pass through the system without the formation of Si, since at the exit valve, a white insoluble material formed in copious amounts. On account of these results, all other experiments were carried out with an argon flow that lay well between 1.0 and 0.1 L/min.

**Figure 4:**
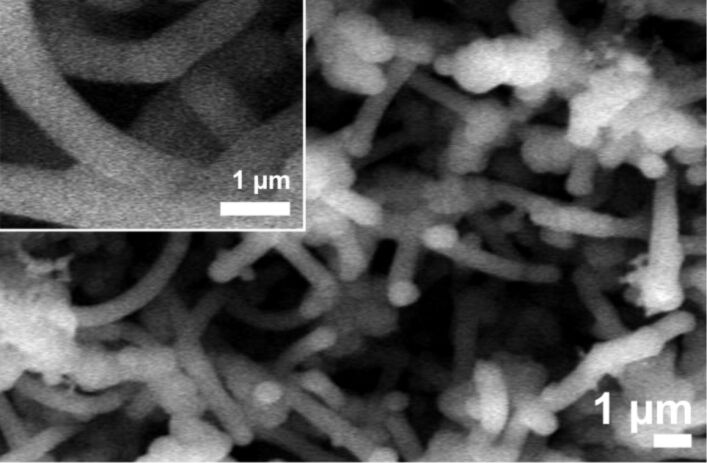
SEM images of NWs grown on gold-sputtered Si[111] by exposure to NPS vapor for 1 h at 650 °C.

The reaction was repeated at 650 °C, but otherwise under the same conditions. SEM measurements of the formed NWs showed similarly shaped growth patterns as for the NWs formed at 375 °C (Figure 4). However, with diameters of about 650 nm, the NWs were slightly thinner than in the previous experiments. One possible reason for this could be that at temperatures as high as 650 °C the sputtered gold layer disrupts to form smaller particles, enhancing the NW growth. Raising of the reaction temperature to as high as 900 °C resulted in the deposition of amorphous silicon throughout the reactor.

### Sputtering and in situ formation of nanoparticles by dewetting

To produce more uniform layers of low diameter NWs, the sputtered Au layers were transformed into nanoparticles before the Si deposition. The amount of gold on the substrate surface was controlled by varying the sputtering time to obtain differently sized gold nanoparticles. Sputtering times were 30 s (sample 1), 1 min (sample 2) and 2 min (sample 3). By means of AFM measurements, we determined the deposition rate to be about 10 nm/min.

**Figure 5:**
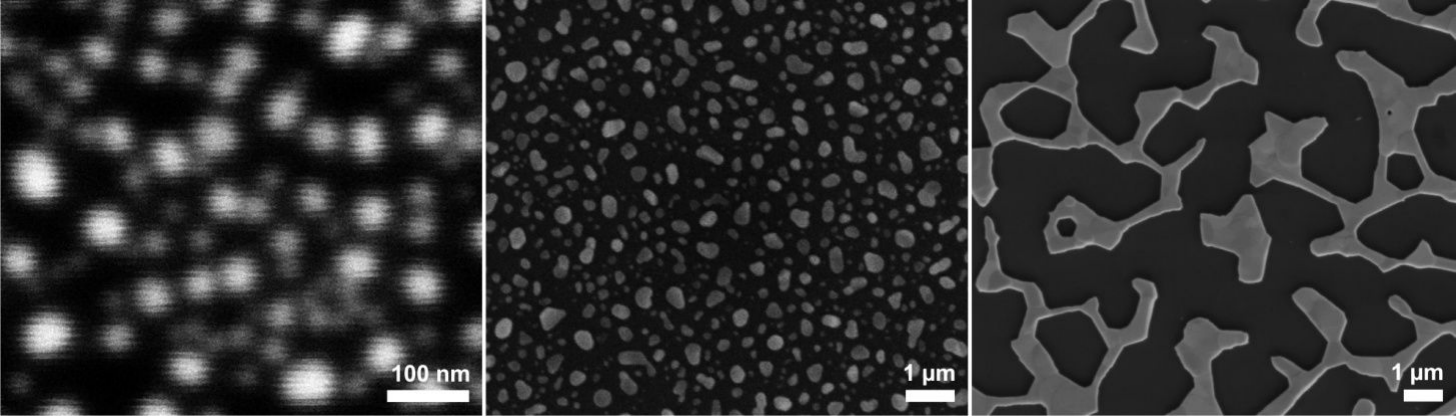
SEM image of gold nanoparticles formed on the native oxide surface of Si[111] after annealing of Au films deposited by sputtering for different lengths of time (left: 30 s; center: 1 min; right: 2 min). The annealing was performed at 650 °C for 1 h.

Upon heating these films to 375 °C no obvious changes occurred, while at 650 °C nanostructures formed within 1 h as a result of a dewetting process [28]. As visible in the SEM images (Figure 5), the size and shape of the nanostructures formed on the three kinds of samples varied considerably. While on samples 1 and 2 separate nanoparticles with diameters of 30–100 nm (sample 1) and 170–300 nm (sample 2) were found, sample 3 rather showed a network of gold after annealing (Figure 5, right).

**Figure 6:**
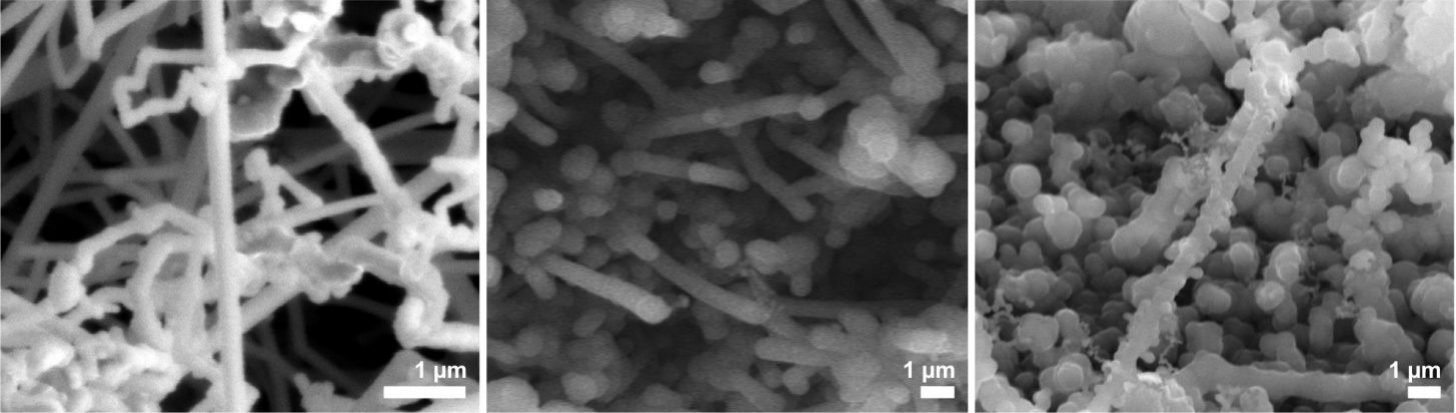
SEM image of NWs grown at 650 °C for 1 h on Si[111] surfaces with annealed gold films (left: sample 1a; center: sample 2a; right: sample 3a).

When these substrates were treated with NPS vapor for 1 h at 650 °C (treatment “a”), the resulting NWs on the three samples also differed in their shapes and diameters. On sample 1a, a dense layer of long NWs with diameters varying between 100 and 200 nm could be observed (Figure 6, left). Again, the NWs were buckled, as in the case of nonannealed Au layers. Additionally, bulkier structures and a few thicker NWs could be observed on the substrate surface. The NWs on sample 2a grew in a more straight fashion with a diameter of 600–700 nm (Figure 6, center). In both cases the diameters of all NWs were more than twice the size of the nanoparticles. The NWs on sample 3a, on which no nanoparticles had been formed during the annealing step, were even bulkier than the ones on the other samples, with a diameter of around 800 nm or more (Figure 6, right). Remarkably, these NWs showed significant branching.

**Figure 7:**
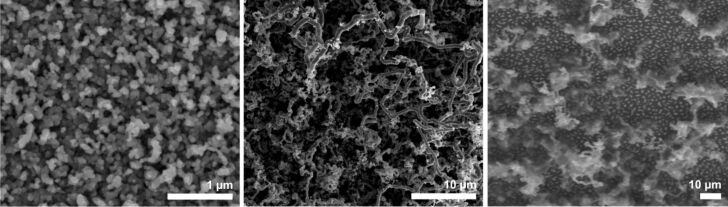
SEM image of NWs grown at 375 °C for 1 h, on annealed gold films of different sputtering times (left: sample 1b; center: sample 2b; right: sample 3b).

Since NW growth was achieved at temperatures as low as 375 °C on sputtered surfaces, the NW growth was repeated at this temperature for 1 h (treatment ”b”) by using again gold films previously annealed at 650 °C for 1 h, as described above. SEM measurements showed the growth of NWs with a diameter of 60–100 nm on sample 1b (Figure 7, left). The NWs have a lot of kinks and are clustered together forming a dense layer on the substrate. On sample 2b, the NWs can be seen more clearly (Figure 7, center). Their buckled growth resembles the NW formation on nonannealed substrates, while their diameter is somewhat smaller, between 300 and 500 nm. On sample 3b, the networked structure of the annealed gold layer is basically maintained. Structures with a size of several micrometers formed on the surface of the substrate. On top of those formations, agglomerated NWs with a diameter of 1 µm started to grow. In between these structures, the formation of particles with a size of around 500 nm could be observed. These particles contained silicon and a high amount of gold, as determined by EDX measurements. No NW growth could be observed from these particles (Figure 7, right). At both temperatures, the diameter of the NWs exceeded the diameters of the catalytically active nanoparticles. Nevertheless, when the growth was performed at 375 °C, the diameter of the NWs was significantly smaller than the diameter of the NWs grown at 650 °C (Table 1).

**Table 1:** Size distribution of the NWs obtained at different temperatures, grown on sputtered gold films of different thickness that had been annealed at 650 °C before the NW formation.

sample	sputtering time	nanoparticles	NWs at 375 °C	NWs at 650 °C

1	30 sec	30–100 nm	60–100 nm	100–200 nm
2	1 min	170–300 nm	300–500 nm	600–700 nm
3	2 min	—	1000 nm	>800 nm

We assume that the nanoparticles become larger by taking up the silicon atoms from the precursor, which results in thicker nanowires. At higher temperatures, the alloy can take up more silicon, resulting in even bigger nanoparticles, further increasing the diameter of the extruded NWs.

### Deposition of preformed nanoparticles from solution

**Figure 8:**
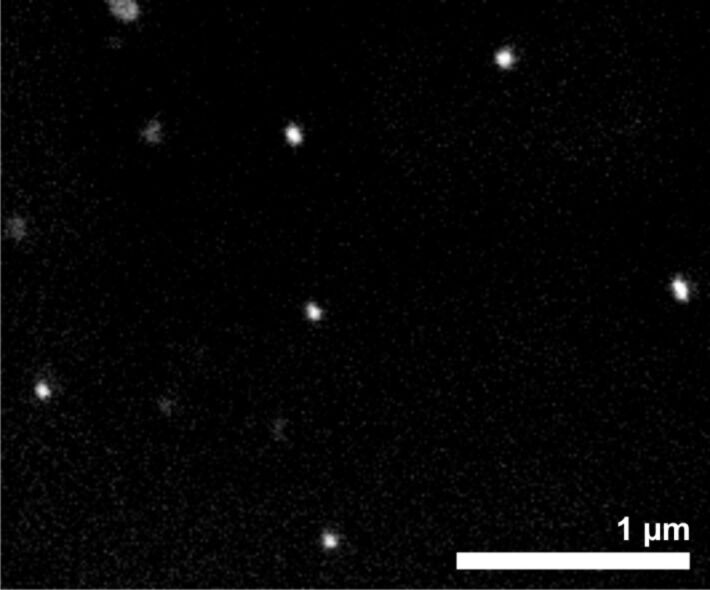
SEM image of the chemisorbed gold nanoparticles on the aminopropylated Si[111] surface.

To decrease the diameter of the NWs, gold nanoparticles with a size of 60 nm were synthesized by following standard protocols [42] and deposited from solution onto Si[111] substrates. For this, the native oxide layer of the silicon wafers was modified by a monolayer of 3-aminopropyl-terminated siloxane [43], the amino groups of which are able to coordinate to the Au nanoparticles. The chemisorption of the nanoparticles proceeded by simple immersion into the respective solution and resulted in surfaces that were evenly, but not closely decorated by the nanoparticles (Figure 8). The average distance between two nanoparticles could be estimated to be about 1 µm.

**Figure 9:**
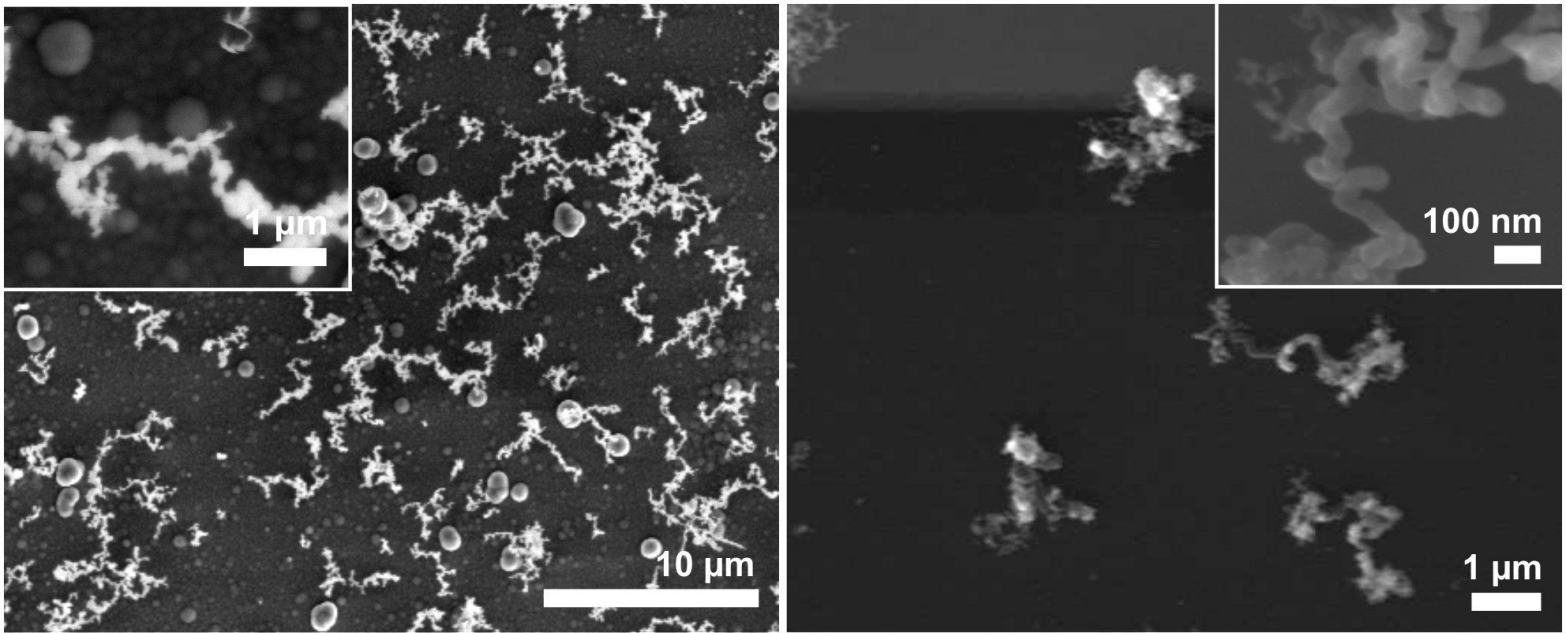
SEM image of NWs grown from nanoparticles at 650 °C without prior treatment (left) and at 375 °C after H_2_-plasma treatment for 40 min (right). Observe that at the lower temperature no round Si particles could be found on the sample.

Using these surfaces without further treatment, no NW growth was observed below 650 °C. At this temperature, NWs with a diameter of less than 100 nm and a length of up to 10 µm formed on the silicon surface (Figure 9, left). We assumed that at this temperature, the organic stabilizers thermally decomposed, making the surface of the nanoparticles accessible for the precursor. To cross-check this hypothesis, we used an alternative, but established protocol [44], i.e., the removal of organic material by a treatment with H_2_ plasma prior to NW deposition. Indeed, after this treatment, the formation of NWs with a diameter of 60 nm and a length of up to 10 µm could be observed already at reaction temperatures of 375 °C (Figure 9, right). Due to the relatively large distance between the nanoparticles, the NWs did not form a dense carpet on the substrate, as was the case with the sputtered substrates. The buckling of the NWs even when growing far apart demonstrates that the buckling does not arise from contact/steric hindrance within the more densely packed NW layers. While the images of the two different methods look quite similar at first sight, it should be mentioned that the pretreatment with H_2_ plasma and the low temperature seems to avoid the formation of the silicon nanoparticles that can be found in between the NWs on the sample formed at 650 °C.

### Nanoparticles from “liquid bright gold” as precursor

One alternative method for the deposition of thin gold films to surfaces is the use of “liquid bright gold”, also called “porcelain gold” or “gold ink”. This material, which is formed by heating gold dust with sulfur and terpenes, was commonly used in the manufacturing and refinement process of porcelain [45]. When the tar-like material is painted onto ceramic surfaces, it can be converted to gold films by simple heating in air. Today it is typically applied by screen printing for the decoration of porcelain and glassware [46].

**Figure 10:**
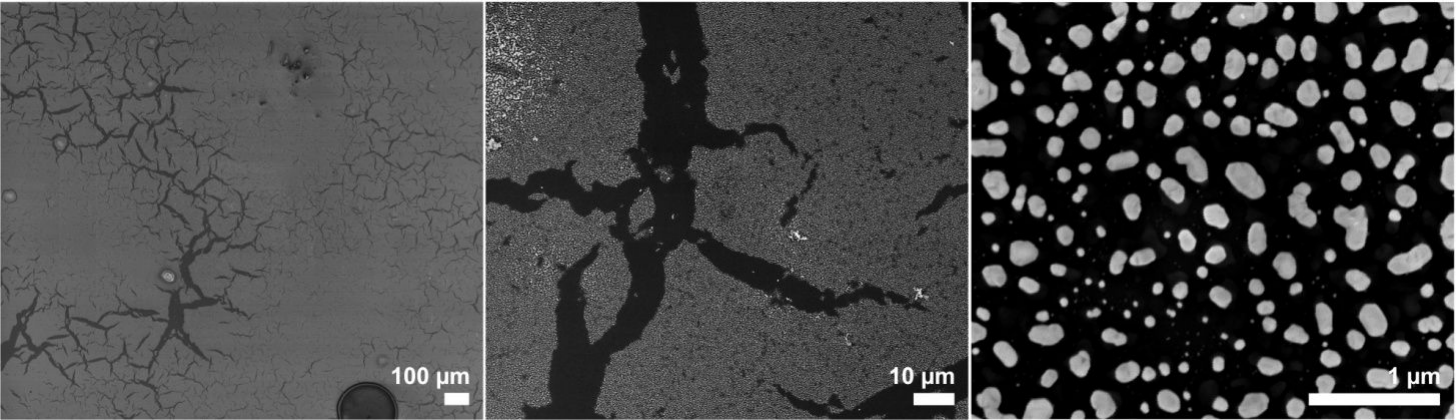
SEM images with different magnifications of a spin coated film of “liquid bright gold” on Si[111] after annealing at 650 °C for 1 h.

To obtain thin gold films, commercial Screen Printing Brightgold 14603 (Surcotech) was diluted with dichloromethane and spin coated onto the native oxide layer of a Si[111] substrate. At 7000 rpm a dense coating was achieved in the form of a sticky film. Annealing of this film at 650 °C for 1 h in the presence of air resulted in dense but inhomogeneous gold particle coverage. Those particles partially resembled the nanoparticle assemblies obtained from the annealing of the sputtered gold films, although their size and shape distribution was much wider (Figure 10, right). Many disruptions as well as areas with more aggregated particles could be found on the substrate surface, which is presumably due to inhomogeneous drying/decomposition of the films during its thermolysis (Figure 10, left and center).

**Figure 11:**
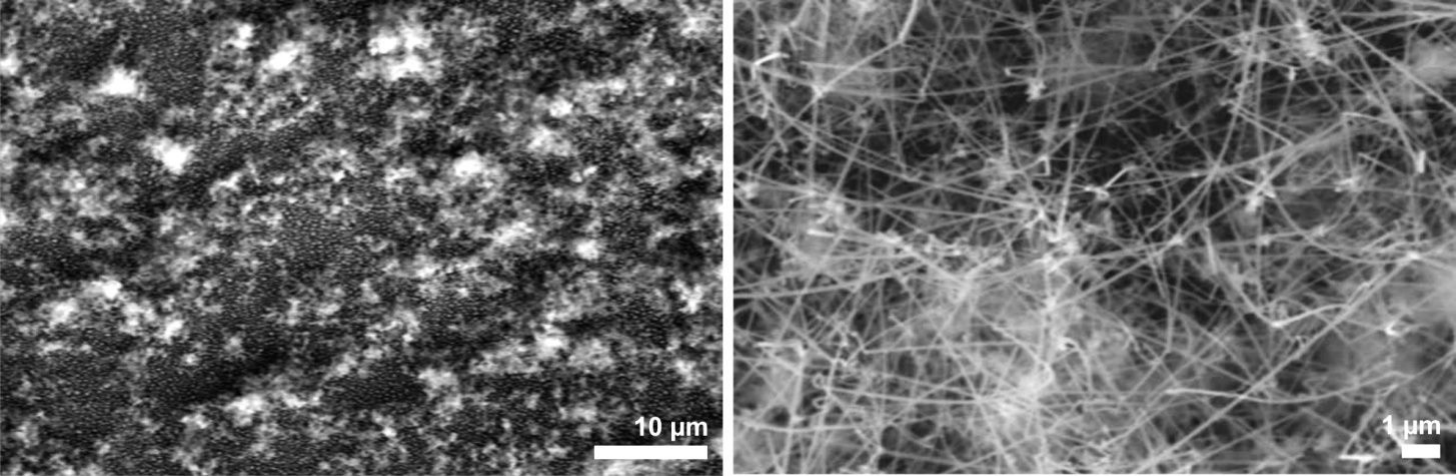
SEM image of Si NWs obtained from spin-coated “liquid bright gold” films after annealing at 650 °C, followed by treatment with NPS at 375 °C (left) and 650 °C (right) for 1 h each.

Again, NW growth was achieved by treatment with NPS at different deposition temperatures. At 375 °C NW growth could be observed, but the NWs were less densely packed than in the sputtered cases. In the background small particles could be found, which contained larger amounts of gold as shown by EDX measurements. The NWs were thin with many kinks, growing on the substrate in an insular mode (Figure 11, left). At 650 °C and a deposition time of 1 h the observed NWs seemed to grow in a straighter fashion with a length of several 100 µm and a diameter of around 150 nm. Additionally, smaller-scaled NWs growing in a buckled way could be observed on the substrate (Figure 11, right).

**Figure 12:**
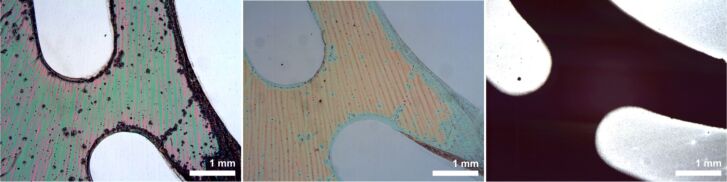
Optical microscopy images of the different steps of the irradiation-induced pattern formation. Left: The “liquid bright gold” film after UV irradiation and washing with dichloromethane. Center: The same area after annealing at 650 °C. Right: The pattern is reproduced after 1 h of treatment with NPS at 650 °C. The bright areas are due to the high reflectivity of the uncovered Si substrate.

**Figure 13:**
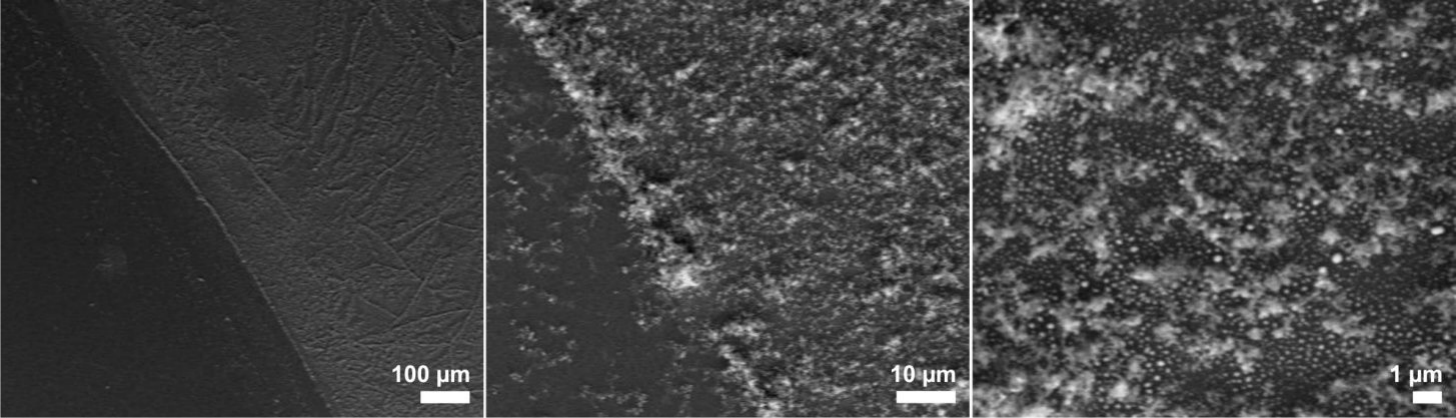
SEM images (different magnifications) of Si NWs obtained from UV-patterned spin-coated “liquid bright gold” films after annealing at 650 °C, followed by treatment with NPS at 375 °C for 1 h.

The polymeric nature of “liquid bright gold” offers opportunities for patterning and hence controlling the growth areas of the NWs. One well-established method for the patterned deposition of polymer films is microcontact printing [47], which is generally considered advantageous since it is an “additive” method. In this particular case the need for dichloromethane or a similar solvent makes this process unsuitable due to the well-known swelling of the stamp material with these solvents [48]. We figured that the gold in these resins might be reducible to the elemental state by irradiation and devised, therefore, a scheme similar to the well-established photolithographic process, which by definition is a “subtractive” one. When a spin-coated layer of the “liquid bright gold” was illuminated with a mercury-vapor lamp through a mask consisting of a metal pattern on a planar quartz substrate, no visible changes took place in the polymer layer. Nevertheless, after washing with dichloromethane, the nonirradiated areas were dissolved, while the irradiated parts of the “liquid bright gold” persisted, reproducing the pattern of the mask (Figure 12, left). Obviously cross-linking occurs during the irradiation, decreasing the solubility of this material (negative photoresist). Annealing of this patterned film for one hour in the presence of air, as described above, left a thin but visible layer of gold with the same pattern (Figure 12, center). The gold-patterned substrates were placed into the reactor and treated with NPS for 1 h at 650 °C (Figure 12, right) and 375 °C. NW growth could be observed in both cases; however, the coverage of the substrate at 375 °C turned out to be not dense enough to form a satisfying pattern (Figure 13).

**Figure 14:**
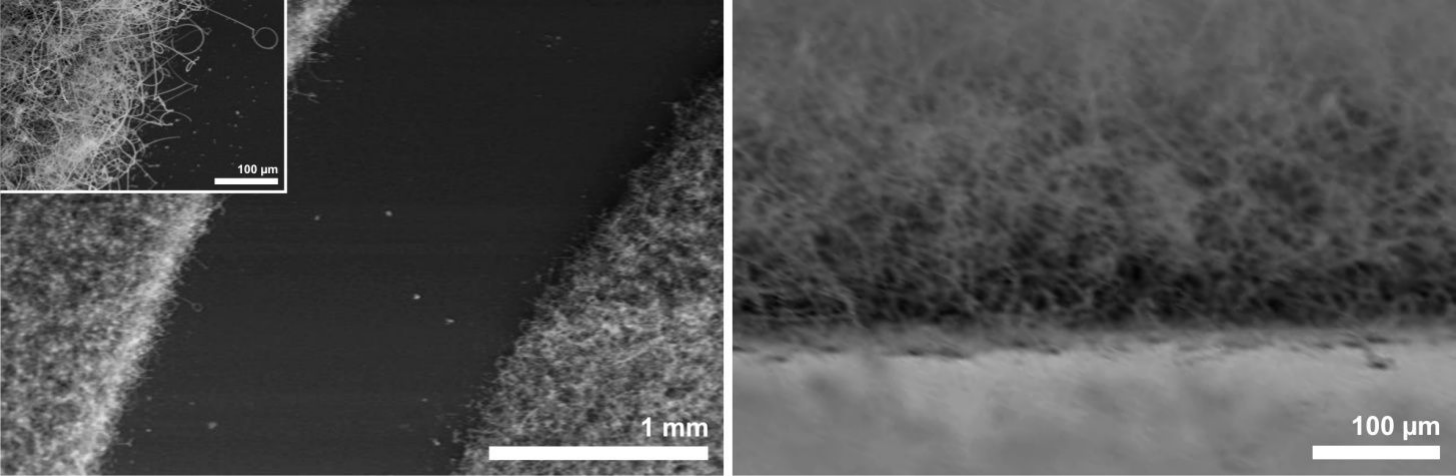
SEM image of the border region of a Si NW pattern obtained from UV-patterned “liquid bright gold” films after annealing at 650 °C, followed by treatment with NPS at 650 °C. Left: Top view, right: Side view.

At a reaction temperature of 650 °C the pattern was much more pronounced. SEM images of the substrate showed that long NWs grew within the previously irradiated areas, similar to those in Figure 11, right. These NWs have a diameter of around 800 nm and grow in a straight fashion over several micrometers (Figure 14). Although the borders of the pattern were relatively sharp, a zone about 100 µm wide could be observed in which the NW growth differed from inner parts of the NW-covered areas. The NWs at the border zone had a diameter of up to 2 µm and grew longer than on the rest of the pattern (Figure 14, left, insert). It remains unclear, why these zones of different morphology are formed in the first place. Currently, investigations are under way to understand this peculiar behavior.

## Conclusion

In this project, we demonstrated that silicon NWs can be reliably prepared from the silicon-rich precursor neopentasilane (Si(SiH_3_)_4_) using gold as catalyst. It could be demonstrated clearly that the formation of the NWs proceeds by the VLS mechanism, in which an Au/Si alloy forms that upon further exposure to the precursor starts excreting the excess silicon in the form of nanowires. The diameter of the nanowires depends on the size of the Au/Si droplets, which in turn can be determined by the size of the employed gold nanoparticles. As a rule of thumb, the diameter of the NWs deposited at 375 °C is about 1.5 times larger than the diameter of the nanoparticles; at 650 °C this factor amounts to ≈2.5, presumably due to the higher solubility of silicon in the Au/Si alloy at higher temperatures.

Following this trend, the thinnest NWs were obtained by using preformed nanoparticles, which became deposited at the substrate surface by chemical means. The fact that the resulting, spatially separated NWs are buckled demonstrates that the shape is not influenced by steric effects but rather by irregularities of the nanoparticles themselves. Furthermore, the nanoparticles had to be deprotected (either by H_2_ plasma treatment or thermal decomposition) to achieve the NW formation. No clear trends could be observed yet regarding the length and the shape (straight/buckled) of the NWs.

In extension of this, a rather classical method for the deposition of decorative gold films was modified for the deposition of gold nanoparticles. Upon annealing of spin-coated films of “liquid bright gold” at 650 °C, dense layers of nanoparticles can be easily formed, which serve as efficient catalysts for Si NW production. An interesting extension of this approach is that these films can be cross-linked by UV irradiation, permitting their lateral patterning. These patterns are maintained during the annealing and the Si deposition steps, so that spatially localized Si NW formation can be achieved.

We believe that the combination of using neopentasilane, as a convenient-to-handle Si precursor, with the different possibilities of gold nanoparticle deposition provides a powerful tool for the fabrication of Si NWs for different applications, such as sensing or photovoltaics. Future work will not only go deeper into determining the guiding principles of NW formation but also in studying the optical and electronic properties of these interesting materials.

## Experimental

All substrates were cleaned with freshly prepared Caro’s acid (consisting of 3 parts of H_2_SO_4_ and 1 part of 30% H_2_O_2_) prior to use. After washing with copious amounts of demineralized water, they were dried in a stream of nitrogen.

Sputtering was carried out for 30 s, 1 min or 2 min by using a Sputter coater S150B from Edwards with argon as the collision gas. For the formation of nanoparticles, the substrates were annealed at 650 °C for 1 h in air. Citrate-stabilized gold nanoparticles were synthesized by the method of Frens [42]. SEM measurements as well as UV–vis spectra [49] indicated a size distribution of around 60 nm. To attach the nanoparticles to the surface, the Si substrates were treated for 2 h with a solution of 10% 3-aminopropyltriethoxysilane in toluene at 100 °C [43]. After washing and sonication of the substrates with ethanol, the nanoparticles were brought to the surface by immersing the aminopropylated substrates into the nanoparticle solution for 90 min. After drying of the substrates in a stream of nitrogen, one group of samples was treated with H_2_ plasma for 45 min, while the other group experienced no further treatment.

For the “liquid bright gold” films, 10% solutions of Screen Printing Bright Gold 14603 (Surcotech) in dichloromethane were filtered (0.2 µm) and spin-coated onto the Si wafers at 7000 rpm. To pattern these films, they were irradiated through a mask (a patterned gold film on a quartz plate), which was placed into direct contact with the film. The best results were obtained after 3 h with a medium-pressure mercury-vapor lamp (25 W). To develop the pattern, the films were purged with dichloromethane. Patterned as well as uniform films were then annealed at 650 °C for 1 h in the presence of air to obtain the Au nanoparticle deposits.

NPS was synthesized by following a literature procedure [50]. For the formation of the Si NWs, the substrates were placed in a quartz glass tube (3 cm diameter), which was heated in a tube furnace to the appropriate temperature, in a stream of argon regulated by a pressure control valve. For the deposition, the NPS vapor was carried into the reactor by an argon stream at about 0.5 L/min, by simply bubbling the gas through the liquid while it was kept at 0 °C. The deposition took place at 375 and 650 °C for a typical reaction time of 1 h. Substrates were analyzed by SEM/EDX (Atomica/Amray, 1920 ECO and FEI Nova Nanolab 600), TEM (FEI Tecnai Spirit) and light microscopy (Reichert, Univar) measurements.
